# Tetracycline Resistance Gene Profiles in Red Seabream (*Pagrus major*) Intestine and Rearing Water After Oxytetracycline Administration

**DOI:** 10.3389/fmicb.2020.01764

**Published:** 2020-08-04

**Authors:** Yumiko Obayashi, Aya Kadoya, Naoto Kataoka, Kazuki Kanda, Su-Min Bak, Hisato Iwata, Satoru Suzuki

**Affiliations:** Center for Marine Environmental Studies, Ehime University, Matsuyama, Japan

**Keywords:** aquaculture, tetracycline resistance, red seabream, seawater, intestine

## Abstract

Marine aquaculture fish and the environment are possible hot spots for the maintenance and spread of antibiotic resistance genes (ARGs). We here show the time courses of changes of six tetracycline resistance genes (*tet*) in fish rearing seawater and fish intestine in tank experiments. Experimental tanks were prepared as oxytetracycline (OTC) administration tanks and those without OTC. It was found that *tet*(B), *tet*(M), and *tet*(W) were dominant in seawater among the six *tet* genes. *tet*(B) and *tet*(M) abundances increased immediately after OTC administration, indicating that OTC served as a selective pressure to increase the proportion of *tet*-possessing bacteria. In contrast, the abundance of *tet* genes in the fish intestine did not differ between the with- and without-OTC administration groups, and clearly was not altered by OTC administration. Profile changing of *tet* in seawater and fish intestine did not synchronize. These observations suggested that the dynamics of intestinal *tet*-possessing bacteria do not directly reflect the environment, but reflect selection within the intestine.

## Introduction

The prevalence of antibiotic-resistant bacteria (ARB) and antibiotic resistance genes (ARGs) has been expanding globally, leading to concern regarding increasing risks to human health (Llor and Bjerrum, 2014). It is well known that the heavy use of antibiotics and synthetic antimicrobials creates a selective pressure on bacteria. ARGs develop in such “hot spots” under selection pressure, in which not only human and veterinary clinics, but also aquaculture facilities are the sites of selection (Cebello, 2006).

Oxytetracycline (OTC) has been used historically in aquaculture in many countries to control fish diseases (World Health Organization [WHO], 2006; Cebello et al., 2013). Tetracycline resistance genes (members of the *tet* series) have been detected in various sites associated with aquaculture, including (for example) sediments beneath net-pens (Tamminen et al., 2011b) and waters close to fish farms (Kim et al., 2004; Suzuki et al., 2019). Environmental bacteria possessing *tet*(M) have been isolated from seawater and sediments at aquaculture sites where OTC has been administered (Nonaka et al., 2007). However, most ARB research has studied cultured fish pathogenic bacteria (Aoki et al., 1974, 1987; Sørum et al., 1992; Kim et al., 2004; World Health Organization [WHO], 2006; Akinbowale et al., 2007), whereas knowledge regarding commensal bacteria living in the fish body remains limited. To date, the results of studies at aquaculture sites have indicated primarily that *tet* genes are distributed in seawater and sediment, reflecting transmission between fish and the environment. We recently reported the detection of *tet*(M) in aquaculture seawater, although the occurrence of this marker varied over the course of a year (Suzuki et al., 2019). Using resistome analysis, Muziasari et al. (2017) reported that intestinal DNA from farm-raised salmonid fish contained high abundances of *tet*(M). Thus, the literature suggests that ARG dynamics relate to aquaculture activities and the fish under study.

We hypothesized that the intestine of farmed fish is an ARG-enhancing spot and serves as the source of the ARGs detected in environmental seawater. To evaluate the risk to humans of ARB and ARG acquisition from edible fish, knowledge of the ARG dynamic state in fish and the environment is needed.

Salmonids are the major marine aquaculture fish species in North and South America and in Europe (FAO, 2010), whereas members of the order Perciformes are the predominant farmed fish in Japan. Aquaculture of rock fish and seabream, a member of the order Perciformes, is increasing in North America and along the Mediterranean coast. ARB studies in the Perciformes are available, although such studies typically are restricted to investigations of isolated fish pathogens (Aoki et al., 1974; Chopra and Roberts, 2001). Recent molecular biological studies have characterized the resistome (Muziasari et al., 2017) and intestinal microfloral metagenomes (Gajardo et al., 2016; Zarkasi et al., 2016), exclusively in salmonid fishes. Culture-independent approaches to characterizing the microbes of Perciformes fish have not been published to date. Anatomical structures of visceral organs and feeding habits differ among various taxa of fish; therefore, the intestinal structures and microflora also are expected to differ among such taxa. Evidence of ARG and microflora changes in fish intestine in aquaculture fish are needed to evaluate antibiotic effects and risks in coastal seas.

The present study sought to reveal a time course of changes in ARB and ARGs in red seabream (*Pagrus major*), a major aquaculture fish of the order Perciformes. Specifically, we examined the state of *tet*-series genes in the intestine and rearing water following the administration of OTC. To our knowledge, this work represents the first experimental study examining changes in *tet* gene occurrence in marine fish intestine and environmental seawater under conditions of drug administration.

## Materials and Methods

### Experimental Administration of Oxytetracycline (OTC) to Red Seabream

**FIGURE 1:**
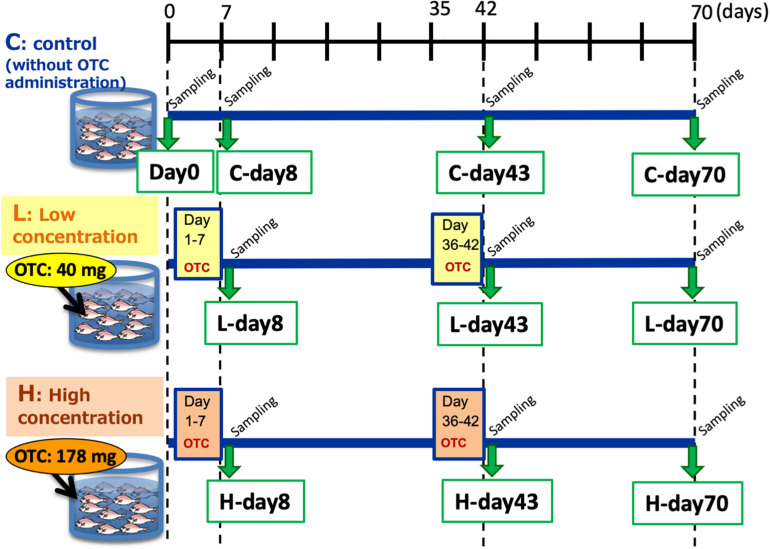
Experimental groups and sampling schedule.

Two-month-old juvenile red seabreams (*P. major*) with average body length of 15 cm were used for the experiment. Fish were obtained from a breeder and acclimated in the experimental tanks without any antibiotics for 2 months prior to the start of the experiment. To initiate the experiment, 50 fish were distributed to each of three tanks (volume 1000 L, Supplementary Figure S1): Control tank without OTC administration, Low-OTC tank with 40 mg OTC (g fish body weight^–1^ day^–1^, equivalent dose to actual aquaculture use) administration, and High-OTC tank with 178 mg OTC (g fish body weight^–1^ day^–1^) administration. Fish were fed once a day (1.5% total weight during acclimation and 2% total weight during the experiment) with commercial pellets. For drug administration, OTC was adsorbed into the feed pellets, which then were coated with oil to prevent dissolution. This oil-coating is general procedure in real aquaculture. The feed pellets for the control tank were prepared by the same procedure without OTC. The experimental schedule is shown in Figure 1. OTC was administered from day 1 to day 7 (the first OTC administration period) and again from day 36 to day 42 (the second OTC administration period, following a 4-week wash-out interval). Following the second OTC administration, the fish were reared without further OTC exposure through day 70 (i.e., 4 weeks after the end of the second OTC administration period).

Seawater for rearing was continuously pumped from the port near the laboratory and pre-filtered (sequentially through sand at 10 μm and 1 μm) prior to flow into the experimental tanks at a flow rate of 6 L min^–1^. The tank-volume of water was replaced more than 8 times per day. Salinity, water temperature, pH, and dissolved oxygen of the water in the tanks ranged between 29.0 and 32.5‰, 23.9 and 26.9°C, 7.72 and 8.13, and 85 and 100%, respectively, throughout the experiment; differences in these parameters among the three tanks were negligible. Fish were reared under a light/dark cycle of 14 h/10 h. Mean fish body length and body weight were 14.8 cm and 103.4 g, respectively, at the beginning of the experiment (day 0); at the end of the experiment (day 70), these parameters were 18.7 cm and 218.3 g, respectively.

### Sampling of Fish Intestinal Contents and Rearing Water

Ten fish were sampled at the beginning of the experiment (day 0), and 10 fish were sampled from each of the three tanks on day 8 (i.e., 1 day after the end of the first OTC administration period), day 43 (i.e., 1 day after the end of the second OTC administration period), and day 70 (i.e., 4 weeks after the end of the second OTC administration period). After dissection of the fish, a segment of the hindgut was recovered and its content was squeezed out and collected into a sterilized tube (Supplementary Figure S1). The collected intestinal contents were immediately frozen at −80°C. The intestinal contents of 4–7 individual fish (from a given sample of 10 fish) from each group were used for this study; the number of fish available at a given time point varied because the sampled fish were shared with another research group.

At the time of each fish sample collection, triplicate 40-mL aliquots of rearing water were obtained from each experimental tank. Each water sample was filtered by passage over a 0.2-μm-pore-size Nucleopore filter to collect bacteria; the resulting filter samples were frozen at −80°C until DNA extraction.

### DNA Extraction

The succession of *tet* genes in the seabream intestinal contents and the rearing seawater during the experiment was examined. DNA from each intestinal sample (50–100 mg individual^–1^) was extracted using an ISOFECAL kit (Nippon Gene, Tokyo, Japan) according to manufacturer’s instructions. DNA trapped on the filter from seawater was extracted using the method of Suzuki et al. (2018). The recovered DNA was quantified by ultraviolet absorption using a DU640 meter (Beckman Coulter, Orange County, CA, United States), and the quality of the DNA was checked by electrophoresis on a 1.0% agarose gel stained with Gel Red (Fujifilm, Tokyo, Japan).

### Quantitative PCR (qPCR) of 16S rRNA Genes and *tet* Genes

Copy numbers of the six selected tetracycline resistant genes [*tet*(B), *tet*(C), *tet*(E), *tet*(M), *tet*(S), and *tet*(W)] were determined by qPCR and normalized to the quantity of 16S rRNA genes in the respective samples. These six *tet* genes previously have been detected in aquatic environmental bacteria (Furushita et al., 2003; Roberts et al., 2012); notably, *tet*(B) and *tet*(M) were the most frequently detected in past reports. We included three efflux genes [*tet*(B), *tet*(C), and *tet*(E)] and three ribosomal protection genes [*tet*(M), *tet*(S), and *tet*(W)]. The qPCR was performed in 20-μL reaction mixtures containing 1 μ SsoFast EvaGreen Supermix (Bio-Rad, Hercules, CA, United States), 500 nM each of forward and reverse primers, and template DNA. Primer sets for each target gene are listed in Supplementary Table S1. The qPCR program was run on a CFX96 Real-Time PCR System (Bio-Rad). The program consisted of enzyme activation (95°C 30 s) followed by 40 cycles of denaturation and annealing/extension (95°C 5 s, 62°C 20 s, and 72°C 15 s for *tet*(B) and *tet*(C); 95°C 5 s, 62°C 15 s for *tet*(E); 95°C 10 s, 57°C 20 s for *tet*(M); 95°C 10 s, 60°C 15 s for *tet*(S); 95°C 10 s, 63°C 15 s for *tet*(W); 95°C 5 s, 50°C 10 s for 16S rRNA genes). Melting curves for the amplicons were determined over the range of 60–95°C. The qPCR measurements for each sample were conducted in triplicate.

### Statistical Analysis and Principal Component Analysis

To test differences in *tet* gene abundances among the sample groups, Tukey-Kramer multiple comparison tests were conducted using total abundance of six measured *tet* genes in each sample. The data for the Tukey-Kramer test (number of the samples, averages and standard deviations in the sample groups) are listed in Supplementary Table S2. To test the hypothesis that the respective *tet* gene is more abundant in the higher OTC-level tank than in the lower OTC-level tank, one-tailed, non-paired Student’s *t*-tests were applied to the rearing water samples. This hypothesis was considered to be true when the *p*-value was smaller than 0.05.

To ordinate the samples by the six *tet* gene profiles, principal component analysis (PCA) was applied. The original data for PCA consisted of the six *tet* gene abundances normalized by the 16S rRNA gene abundance in the respective sample. The analysis was started from the correlation matrix of all the samples including the rearing waters and the fish intestine, and the eigenvalues and eigenvector were calculated based on Jacobi’s method using Visual Basic for Applications (VBA; Microsoft Office).

### Bacterial Community Structure

Succession of the bacterial community structure in the fish intestine and the rearing water during the study interval was investigated by 16S rRNA gene metagenomic sequencing. The extracted DNA samples from individual fish or filters were combined within the same group/date to obtain average profiles. The libraries for sequencing the variable V3–V4 regions of the 16S rRNA genes were prepared using adapter-overhung 341F (5′-CCTACGGGNGGCWGCAG-3′) and 805R (5′-GACTACHVGGGTATCTAATCC-3′) primers (Herlemann et al., 2011), according to the protocol for the preparation of 16S rRNA gene amplicons for the Illumina MiSeq system. Sequencing was conducted on an Illumina MiSeq Next Generation Sequencer, yielding more than 100,000 paired-end reads for each pooled sample. After preprocessing such as removing adapter sequences, trimming of low-quality reads, and paired-read joining, sequencing data were cleaned up and processed for population analysis by QIIME pipeline (ver. 1.8.0) (Caporaso et al., 2010). During the cleanup process, sequences <200 bases or including homopolymers were excluded. Trailing part of N bases or the 50-base average quality score below 25 were removed from the sequences. Operational taxonomic units (OTUs) were determined with a 97% similarity threshold. Rarefaction curves plotting the number of OTUs over the number of sequences are shown in Supplementary Figure S2. The phylogenetic assignment of each OTU was carried out with Greengenes 16S rRNA gene database (version 13_8). The assignment of some major OTUs were rechecked with NCBI database Nucleotide collection.

Bacterial community structures were compared on a similarity dendrogram based on Bray-Curtis similarity matrix. Bray-Curtis similarities were calculated from the relative abundances of 260 OTUs which contribute more than 0.01% of the total reads. The dendrogram was constructed by hierarchial agglomerative methods. The calculation and dendrogram construction were performed by PRIMER v7 (Clarke and Gorley, 2015).

## Results and Discussion

### *tet* Genes in the Rearing Water

**FIGURE 2:**
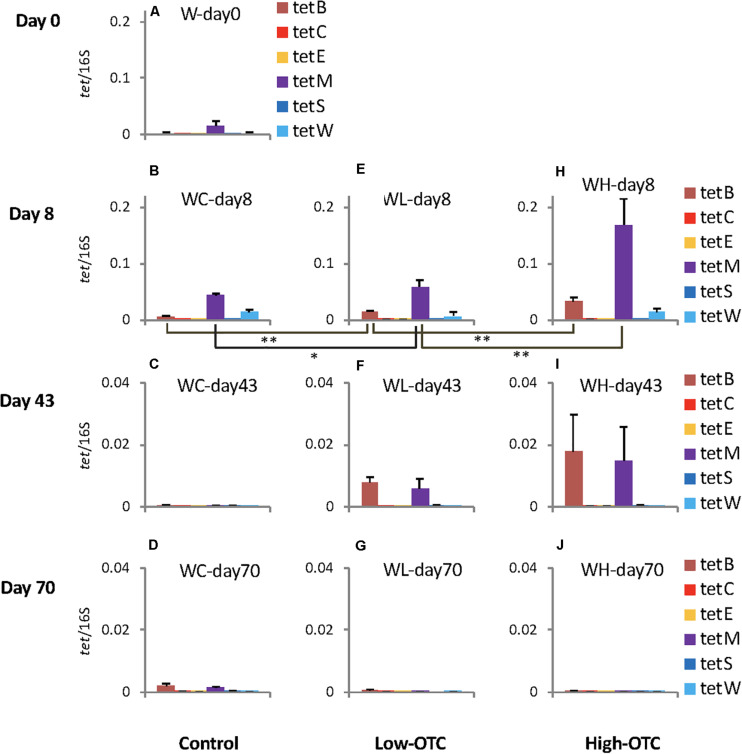
Copy numbers of the six *tet* genes in rearing water in each tank; values were normalized to those of 16S rRNA genes in the respective samples. Error bars indicate the standard deviation of triplicate samples. Single asterisk, *p* < 0.05; double asterisk, *p* < 0.01. In **(A–J)** sample codes are: W, water; C, control; L, Low-OTC; H, High-OTC; and days.

In the control tank without OTC administration, *tet*(B), *tet*(M), and *tet*(W) were detected on days 0 and 8 (Figures 2A,B), but only trace levels could be detected on days 43 and 70 (Figures 2C,D). The water was pumped continuously into the tank, and the tank-water was being replaced more than 8 times per day. The detected amounts of *tet* genes from the rearing water in the control tank could therefore be considered as the background levels for each day, and presumably were derived from a combination of the original seawater inflow and the fish feces. *tet*(B) and *tet*(M) are frequently detected in seawater bacteria (Furushita et al., 2003; Kim et al., 2007; Roberts et al., 2012), and *tet*(W) is known to be present in sediments in rivers and the sea (Suzuki et al., 2008). Notably, *tet*(B) is frequently found in cultured Gram-negative bacteria, and *tet*(M) has the widest host range in bacteria of all *tet* genes (Roberts et al., 2012). The present study confirmed that these genes persist ubiquitously in aquatic environments, which is consistent with the results of previous reports. Similarly, river water inflowing to trout farms has been reported to be a source of contamination by human-derived Salmonella (Antunes et al., 2018). Seawater also has been reported to serve as a reservoir of *Escherichia coli* with ARGs that are carried by seagulls (Alves et al., 2014).

On day 8, following 1 week of OTC administration, copy numbers of *tet*(B) and *tet*(M) differed among the experimental tanks (Figures 2B,E,H), with the levels of both genes being lowest in the control tank. Levels in the Low-OTC tank (Figures 2B,E) were significantly higher than in the control tank [*p* < 0.01 for *tet*(B), *p* < 0.05 for *tet*(M)], and those in the High-OTC tank were significantly higher than in the Low-OTC tank (*p* < 0.01) (Figures 2E,H). Thus, the abundance of *tet*(B) and *tet*(M) in the rearing seawater on day 8 appeared to reflect OTC in a dose-dependent fashion. The levels of *tet*(W) did not differ significantly among the three tanks.

On day 43 (following the second OTC administration period), the copy numbers of *tet*(B) and *tet*(M) in the Low-OTC tank were significantly higher than those in the control tank [*p* < 0.01 for *tet*(B), *p* < 0.05 for *tet*(M)], and the levels of these genes in the High-OTC tank were nominally higher than those in the Low-OTC tanks, although these differences were not statistically significant (Figures 2F,I). At 4 weeks after the end of the second OTC administration period (day 70), differences in the levels of *tet* genes between the Low- and High-OTC tanks were not detected, and the *tet* gene abundances in the tank water did not appear to correlate with OTC level.

These results demonstrated that specific *tet* genes were detected at varying levels in seawater, and that *tet*(B) and *tet*(M) were the most abundant among the six targeted *tet* genes. The levels of these *tet* genes were higher in the OTC-administered tanks immediately after the end of the first OTC administration period, indicating that OTC served as a selective agent for bacteria possessing these *tet* markers.

In previous work, we reported that *tet*(M) was distributed in natural seawater around aquaculture pen-nets, although the occurrence of this gene varied over the course of a year (Suzuki et al., 2019). Transitions in the levels of other *tet* genes in seawater were observed in the present study. Although the cause of the fluctuation of *tet* genes remains unclear, this variability might reflect changes in bacterial numbers. Notably, fish intestinal flora is highly variable (Zarkasi et al., 2016), and fish intestinal flora could be a key source of the environmental flora (Tamminen et al., 2011a). Intestinal community structure did not exhibit drastic variation in the present study, as shown below. However, the abundance of *tet*-possessing bacteria is expected to vary over time.

### *tet* Genes in the Red Seabream Intestine

**FIGURE 3:**
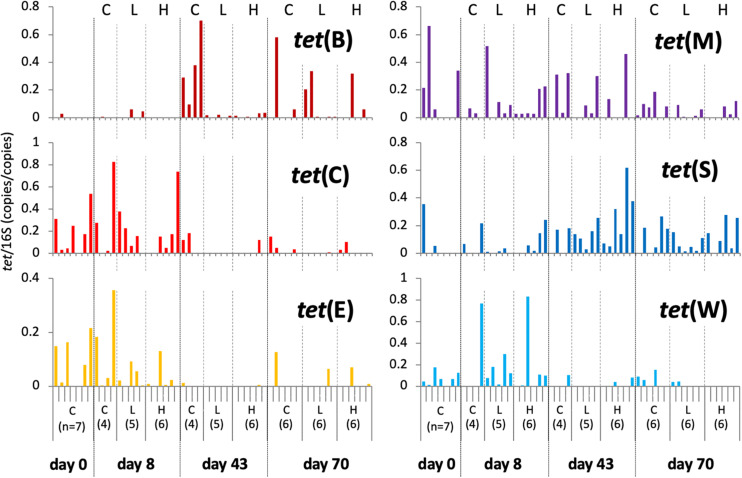
Copy numbers of *tet* genes in fish intestinal content; values were normalized to those of 16S rRNA genes in the respective samples. Results for individual fish are shown separately within each group (*n* = 4–7) and sampling day. C, control; L, Low-OTC; H, High-OTC.

**FIGURE 4:**
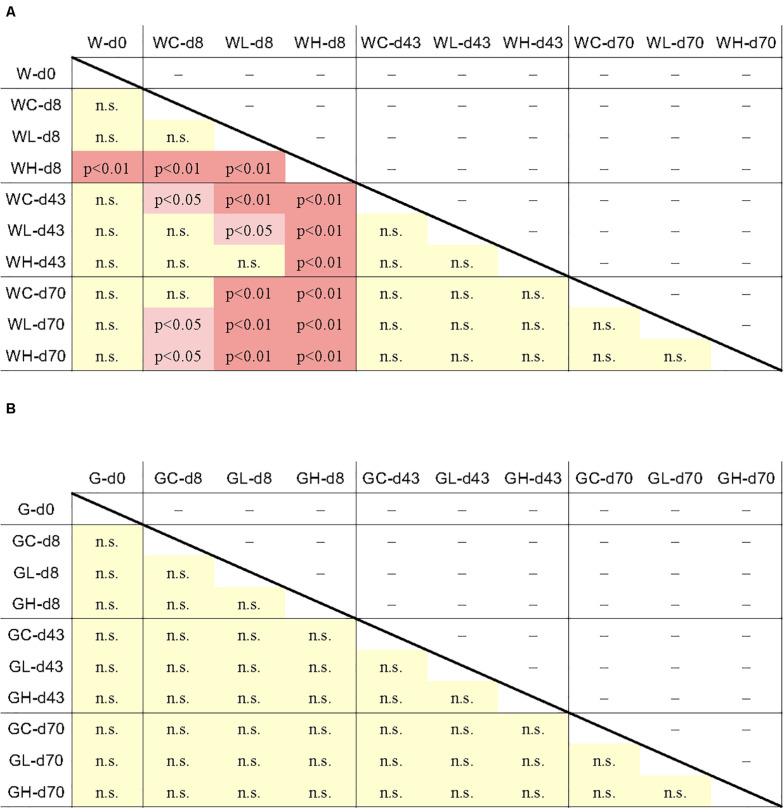
Results of the Tukey-Kramer multiple comparison test for all pairwise comparisons of *tet* gene abundance (six *tet* genes total) between the average of each sample group. Difference of the *tet* gene abundance between the sample groups was significant when *p* < 0.05 or *p* < 0.01. **(A)** rearing water; **(B)** fish intestine. n.s., not significant.

Data from individual fish are shown in Figure 3 and Supplementary Figure S3, and the averages and standard deviations in each sample groups are listed in Supplementary Table S2. Copy numbers of *tet* genes in the fish intestine differed among individuals, even among individuals within the same group/time point (Figure 3). Some individuals in the control tank at the beginning of the experiment (day 0) harbored several *tet* genes (Supplementary Figure S3), indicating that *tet* gene-possessing bacteria were present in the intestine even before experimental OTC administration. All six genes were detected at day 0, suggesting ubiquitous persistence of various *tet* genes. Changes of each *tet* gene abundance in fish during the experimental period were not uniform: *tet*(C), *tet*(E), and *tet*(W) were rather abundant during the earlier period of the experiment (days 0 and 8); *tet*(B) was more abundant during the later period (days 43 and 70); *tet*(M) and *tet*(S) were present through the study period. Total abundance of six *tet* genes in each fish were not significantly different among sample groups (treatment/time) of fish (Figure 4B), not likely as those of the rearing water samples (Figure 4A), suggesting that individual differences were greater than the differences among fish from different tanks (Control, Low-OTC and High-OTC tanks).

Notably, the *tet* profiles in water and intestine did not synchronize, indicating that the environmental *tet* genes do not reflect the intestinal *tet* profiles. This observation suggested that the selection of *tet* genes occurs within the intestine. Rapid water exchange and tight binding of OTC to feed might avoid retaining and spreading of OTC within tank, thus OTC in tank-water did not affect intestinal *tet* genes. It has been reported that the fish intestinal microflora is established through several stages from hatching to adulthood (Hansen and Olafsen, 1999). Diet is an important factor in determining the intestinal microflora (Zarkasi et al., 2016). Wild fish recovered from around OTC-treated aquaculture sites are known to carry resistant bacteria in their intestine (Björklund et al., 1990). The OTC resistance genes are expected to have spread widely over the history of commercial aquaculture, reflecting various factors acting on the fish and on the bacteria, including horizontal gene transfer and competition within the intestinal microfloral community. The observed profiles further showed that individual differences are large, and general tendencies were not apparent even among fish reared under the same conditions. The *tet* genes in individual fish showed variation among similarly treated groups (Supplementary Figure S3). Variation of ARGs in fish intestine previously has been demonstrated using resistome analysis (Muziasari et al., 2017). Further epidemiological or experimental evidence will be needed to clarify issues of *tet* succession and abundance in the fish intestinal community.

Some antibiotics such as sulfonamides had been used frequently in the past in aquaculture, and OTC is still being used in this context; thus, the ARGs for these drugs likely persist in diverse bacterial communities and continue to cycle between the environment and fish. This possibility is supported by research monitoring ARGs at given aquaculture sites over time (Tamminen et al., 2011b; Muziasari et al., 2014, 2017; Suzuki et al., 2019). The results of the present study also suggested that the long history of OTC use along the Japanese coast has maintained a background prevalence of *tet* genes. Fish are expected to continue to be a reservoir for *tet* genes and to play a role in *tet* gene dynamics in seawater.

In conclusion, our examination of *tet* gene succession in fish indicated that the abundance of *tet* genes in the fish intestine did not differ for fish grown with or without OTC, and did not appear to be clearly altered by OTC treatment.

### PCA of the *tet* Gene Profiles

**FIGURE 5:**
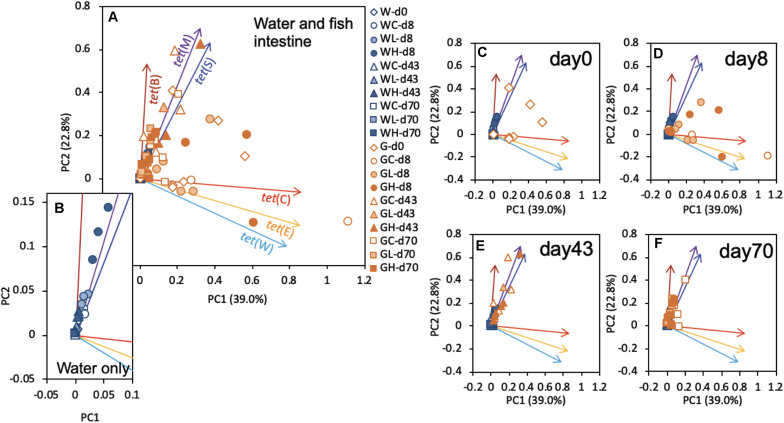
Plots of principal components 1 (PC1) and 2 (PC2) resulting from the principal component analysis (PCA) based on the *tet* gene profiles of all samples. **(A)** Results for all samples are plotted with representative symbols: the initial W or G in the name of each sample indicates whether the sample was obtained from the rearing water or fish gut, respectively; the second character (C,L, or H) indicates whether the sample was obtained from the control, low-OTC, or high-OTC tank, respectively; and the remaining part of each sample name (-dxx) indicates the experimental time point of the sample (where d = day and xx = the day number). The subsequent panels present the same results replotted by extracting only the data for **(B)** water samples, **(C)** water samples and day-0 fish intestine, **(D)** day-8 fish intestine, **(E)** day-43 fish intestine, or **(F)** day-70 fish intestine.

Principal component analysis identified the modalities of gene succession of the six tested *tet* genes, using values normalized to those of the 16S rRNA genes (Figure 5). Although there were not any distinct clusters on the PCA plot for *tet* genes, as shown in Figure 5A, the rearing water samples exhibited a distribution along a line largely superimposable with that of *tet*(M) (Figure 5B). Specific clusters could not be detected in the fish intestine samples on days 0 and 8 (Figures 5C,D). The results on days 43 and 70 exhibited shifts in the direction of *tet*(M) and *tet*(S) (Figures 5E,F). These profiles were similar to those in the respective water samples (Figure 5B). In the human intestine, it is suspected that the intestinal bacteria are able to not only exchange ARGs, but that they are also capable of acquiring and transmitting ARGs from ingested (environmental) bacteria (Sayers et al., 2004). Components of the fish intestinal bacterial community might behave similarly. As mentioned above, *tet*(M) and *tet*(S) were present throughout the study interval, a pattern that was distinct from that of other *tet* genes. *tet*(M) and *tet*(S), which are ribosomal protection protein (RPP) genes, appear to have diverged from the elongation factor-encoding genes in the distant past (Kobayashi et al., 2007), and *tet*(M) is known to be the most widely detected *tet* (Roberts et al., 2012). Plasmid-borne RPP genes are expected to have become widely distributed in the environmental communities due to their greater stability (Bien et al., 2015, 2017). Fish samples showed distinct *tet* profiles that changed with time. The *tet* profile succession would proceed similarly to those of environmental samples and therefore to converge on similar cluster patterns as those seen in seawater.

### Bacterial Community Structure in the Rearing Water and Fish Intestine

**FIGURE 6:**
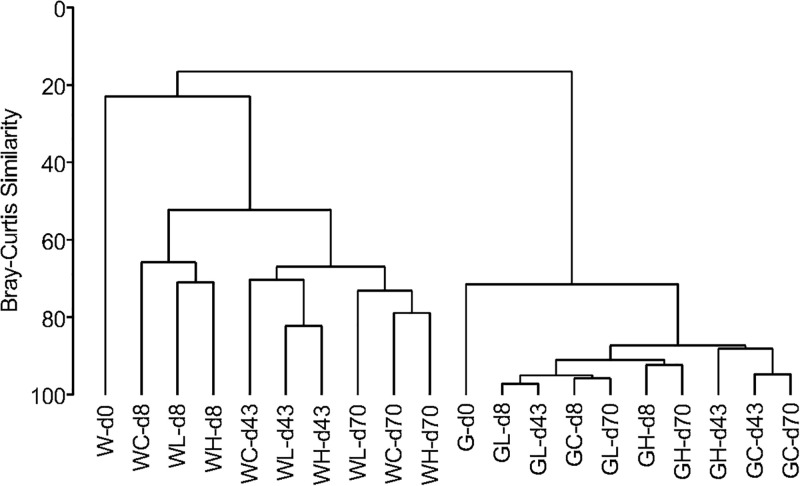
Dendrogram for clustering of microbial communities based on OTUs obtained from metagenomic sequencing of 16S rRNA gene V3–V4 region. Clustering is on the basis of a distance calculated from Bray-Curtis similarities. Sample codes are as follows: W, water samples; G, fish gut (intestine) samples.

A similarity dendrogram showed that microbial communities in the rearing water and in the fish intestine were very different (Figure 6). Communities in rearing waters distributed in groups based on the day of experiment not on the OTC-treatment, indicating that the microbial community structure in the water did not differ among the control, Low-OTC, and High-OTC tanks. Comparing to the microbial communities in the water, those in the fish intestine showed very high similarity throughout the experiment period.

**FIGURE 7:**
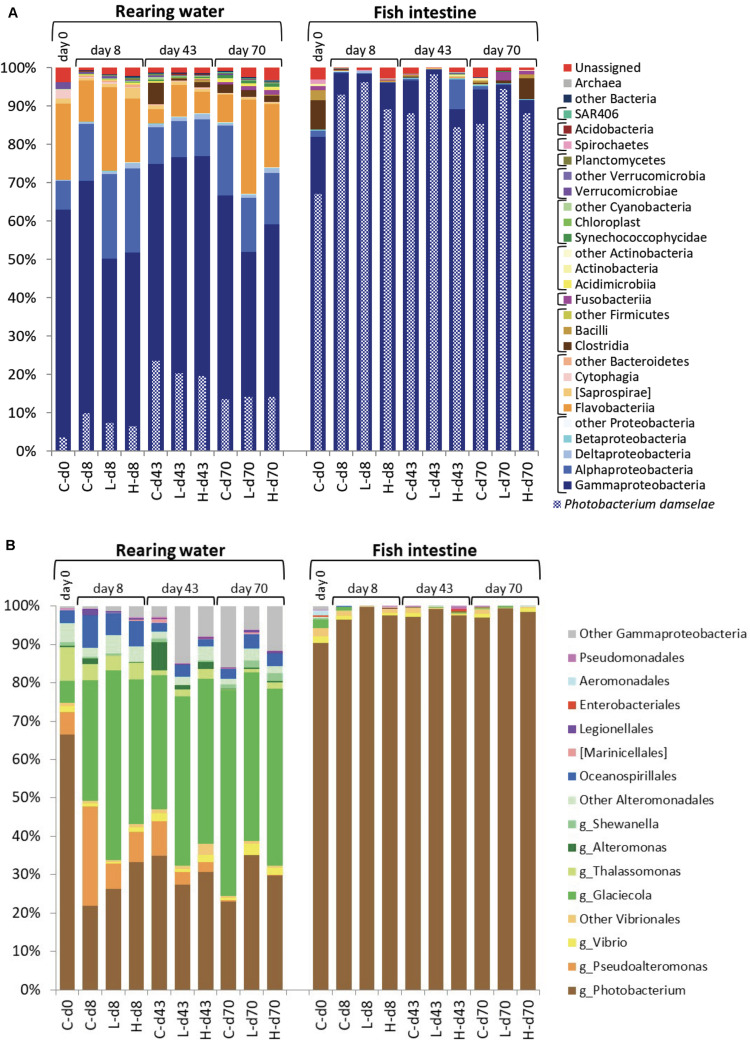
Prokaryotic community structure (abundance rate) in the rearing water and fish intestine. **(A)** Phylum-Class level in the whole community. *Photobacterium damselae* abundances are indicated with checkered bars. **(B)** Order level in the Gammaproteobacteria with some major genera indicated. DNA samples from the same experimental tank on the same day were combined. In the inserted legend, “g_” indicates genus. At the bottom of each panel, the sample names for each bar are coded as follows: C, control; L, low-OTC; H, high-OTC; d and number, days after initiation of study.

Major components of bacterial communities in the rearing water included typical marine bacteria such as members of the classes Gammaproteobacteria, Flavobacteriia, and Alphaproteobacteria (Figure 7A-left). Among Gammaproteobacteria, *Photobacterium damselae* is frequently isolated in the coastal seawater in Japan, and reported to have new plasmids (Nonaka et al., 2012). We thus examined detail species among the Gammaproteobacteria. It was found that the contribution of *P. damselae* to the total population in rearing seawater ranged from 3.6 to 24% over the course of the study interval. In the case of fish intestine, Gammaproteobacteria were detected at high abundance (Figure 7A-right), whereas Flavobacteriia and Alphaproteobacteria were detected at lower abundances than those in the rearing water. Among the Gammaproteobacteria, *P. damselae* was the predominant species (constituting 67% and 84–98% of the total on day 0 and on other days, respectively), regardless of the time point or OTC administration status. Thus, the diversity of bacterial communities in the fish intestine was lower than that in the rearing water throughout the experiment. Together with the facts that the *tet* gene profiles changed over time (Supplementary Figure S3) while the community structures were relatively stable (Figures 7A,B), this observation suggested that *tet*-possessing bacteria within the same clade are not clonal and change over time. The microbial community in the rearing water presumably was formed as a combination of the original seawater community and the intestinal fecal community excreted by the fish.

Bacteria isolated from marine aquaculture environments in Japan, Korea, and China have been shown to possess abundant *tet*(A), *tet*(B), and *tet*(M) genes (Kim et al., 2004; Dang et al., 2007), where the genera *Vibrio* and *Pseudoalteromonas* (both are Gammaproteobacteria) have been reported as reservoirs of the these *tet* genes when bacteria were isolated by culturing. Another report using culturing of bacteria recovered from Chilean salmon also reported that various bacterial isolates possess a diversity of *tet* genes (Miranda et al., 2003). Among Gammaproteobacteria in the present study, *P. damselae* predominated (constituting more than 60% of the Gammaproteobacteria) in seawater at the study start, followed by *Thalassomonas*, *Pseudoalteromonas*, and *Glaciecola* (Figure 7B-left). *Glaciecola* became the predominant genus after day 8. Bacteria of the genus *Vibrio* were detected in all groups throughout the study interval, albeit at very low abundances. Past reports using culture methods showed that *Vibrio* and *Pseudoalteromonas* were the major ARG-possessing genera in seawater (Miranda et al., 2003; Kim et al., 2004; Dang et al., 2007). Use of metagenomic approaches together with culture methods is expected to yield better understanding of bacterial distribution; a focus on culturable bacteria possessing ARGs clearly would provide overestimates of ARG-bearing microbes. Fish intestine samples showed very strong abundance of *Photobacterium*, which in the present study constituted more than 90% of the Gammaproteobacteria throughout the experimental interval (Figure 7B-right). As mentioned above (Figure 7A-right), the species *P. damselae* constituted more than 90% of the fish intestine bacterial population (as judged by metagenomic analysis). Although the intestinal flora differed from the seawater population, the PCA profiles of *tet* genes in the fish intestine on days 43 and 70 were converging with those in the rearing water (Figure 5). These observations again suggested that *tet* genes are possessed by various species and the occurrence dynamics is not a constant tendency in schools of aquaculture fish. The natural background in seawater might contain diverse bacterial species harboring *tet* genes, especially *tet*(M) and *tet*(W), the abundances of which may vary over time.

Based on the results of previous experiments using cultured bacteria, *tet*(M) can be transferred from marine bacteria to human enteric bacteria (Neela et al., 2009; Kohyama and Suzuki, 2019). Kohyama and Suzuki (2019) reported that *P. damselae* transferred *tet*(M)-including multidrug-resistance plasmid to *E. coli* when the donor *P. damselae* was not starved, even in the oligotrophic condition. In general, bacteria in fish intestine are not thought to be starved because of abundant organic matters in the intestine. Combining these previous reports and the results of the present study, it can be supposed that *tet* genes possessed by *P. damselae* in fish feces have potential to be transferred to not only marine bacteria but also enteric bacteria in coastal aquaculture site. Attention will need to be paid to the risk of hidden ARGs in marine microbial communities and environments.

## Conclusion

In conclusion, *tet*(M), *tet*(B), and *tet*(W) were present in seawater, and *tet*(M) and *tet*(B) abundances were increased by OTC administration. The profiles of fish intestinal *tet* genes differed from those in seawater. It was shown that *tet* genes were retained in the fish intestine at 4 weeks after cessation of OTC administration. Metagenomic analyses demonstrated that the microbiomes of seawater and fish intestine differed; notably *P. damselae* was highly abundant in fish intestine. Taken together with previous reports that employed culture methods (Nonaka et al., 2007, 2014), the results of the present study suggested that *P. damselae* may be one of reservoirs of *tet* genes in the coastal marine environment.

## Data Availability Statement

The raw data supporting the conclusions of this article will be made available by the authors, without undue reservation.

## Ethics Statement

This animal study was reviewed and approved by the Animal Experiment Committee of Ehime University.

## Author Contributions

YO: data analysis and draft writing. AK: data analysis. NK: most experiment. KK and S-MB: sampling. HI: fish experiment scheduling. SS: total study design and manuscript writing. All authors contributed to the article and approved the submitted version.

## Conflict of Interest

The authors declare that the research was conducted in the absence of any commercial or financial relationships that could be construed as a potential conflict of interest.

## Supplementary Material

FIGURE S1 Fish rearing system used in this study **(A)**, and dissection used to recover the intestinal content from the fish **(B,C)**.

FIGURE S2 Rarefaction curves plotting the number of OTUs. At the bottom of each panel, the sample names for each bar are coded as follows: W, water samples; G, fish gut (intestine) samples.

FIGURE S3 Copy numbers of *tet* genes in individual fish intestines; values were normalized to 16S rRNA gene copy number in the respective sample. Different *tet* genes are indicated by different bar colors.

TABLE S1 List of primers used in this study for the quantitative PCR targeting of six *tet* genes and the 16S rRNA genes.

TABLE S2 Average and standard deviation of *tet* gene abundances normalized by quantity of 16S rRNA gene (*tet* copies/16S copies). n: Number of the samples in each sample group.
