# Supplementary figures and images for: Tetracycline Resistance Gene Profiles in Red Seabream (Pagrus major) Intestine and Rearing Water After Oxytetracycline Administration
Source: Front Microbiol. 2020 Aug 4;11:1764. doi: 10.3389/fmicb.2020.01764 (PMC7417432; doi:10.3389/fmicb.2020.01764)

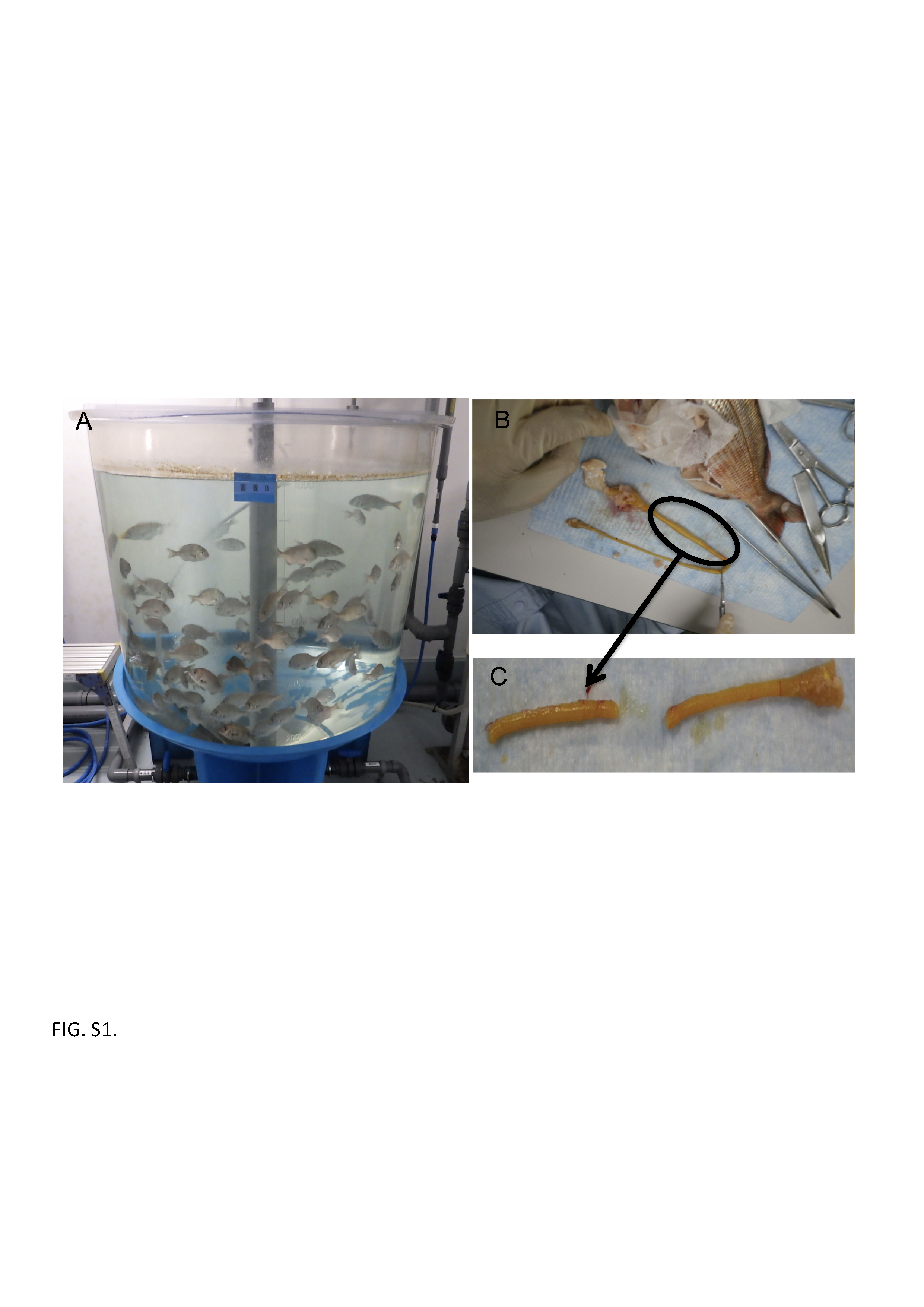

Supplement: FIGURE S1 — Fish rearing system used in this study (A), and dissection used to recover the intestinal content from the fish (B,C). [file Image_1.tiff]

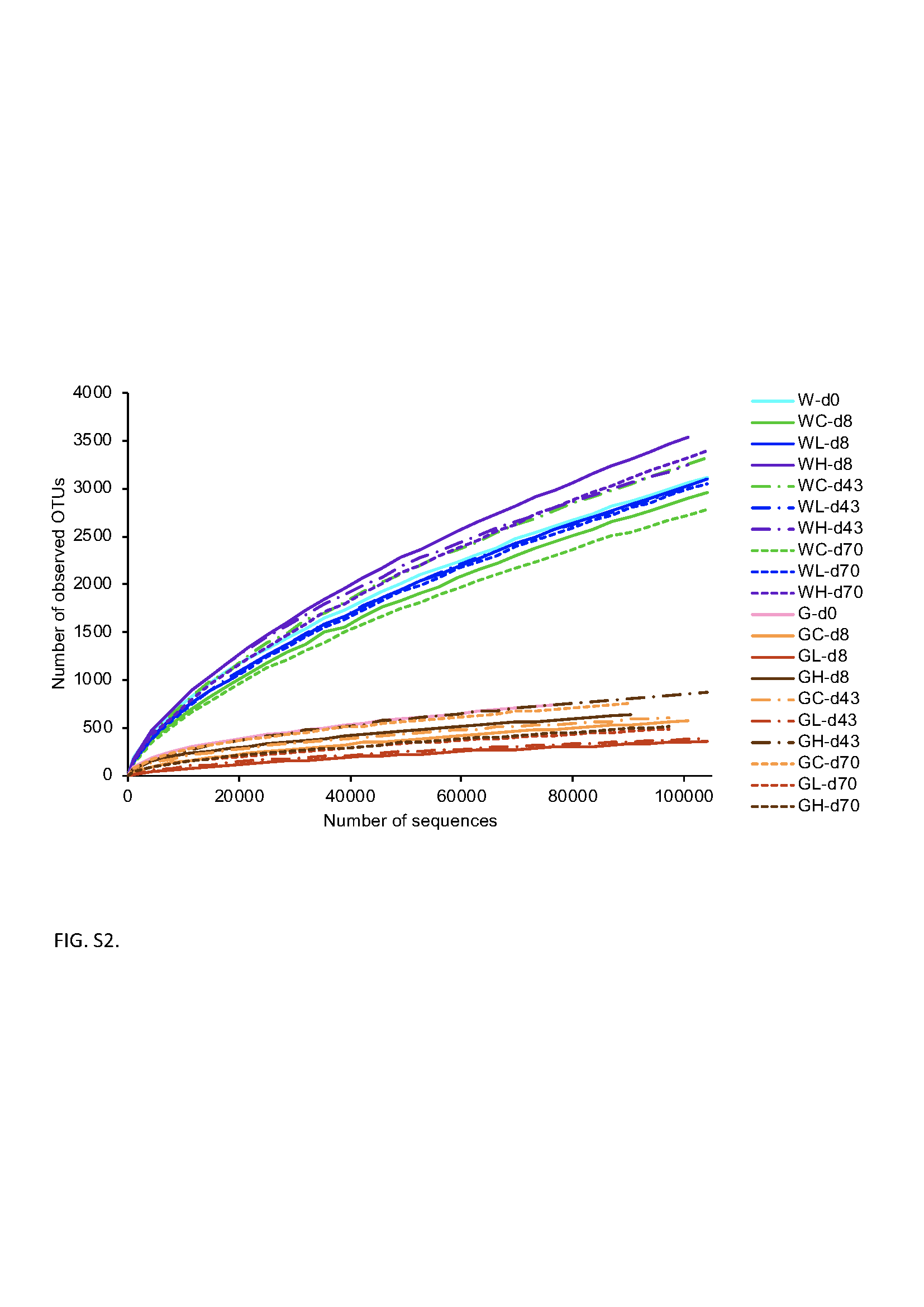

Supplement: FIGURE S2 — Rarefaction curves plotting the number of OTUs. At the bottom of each panel, the sample names for each bar are coded as follows: W, water samples; G, fish gut (intestine) samples. [file Image_2.tiff]

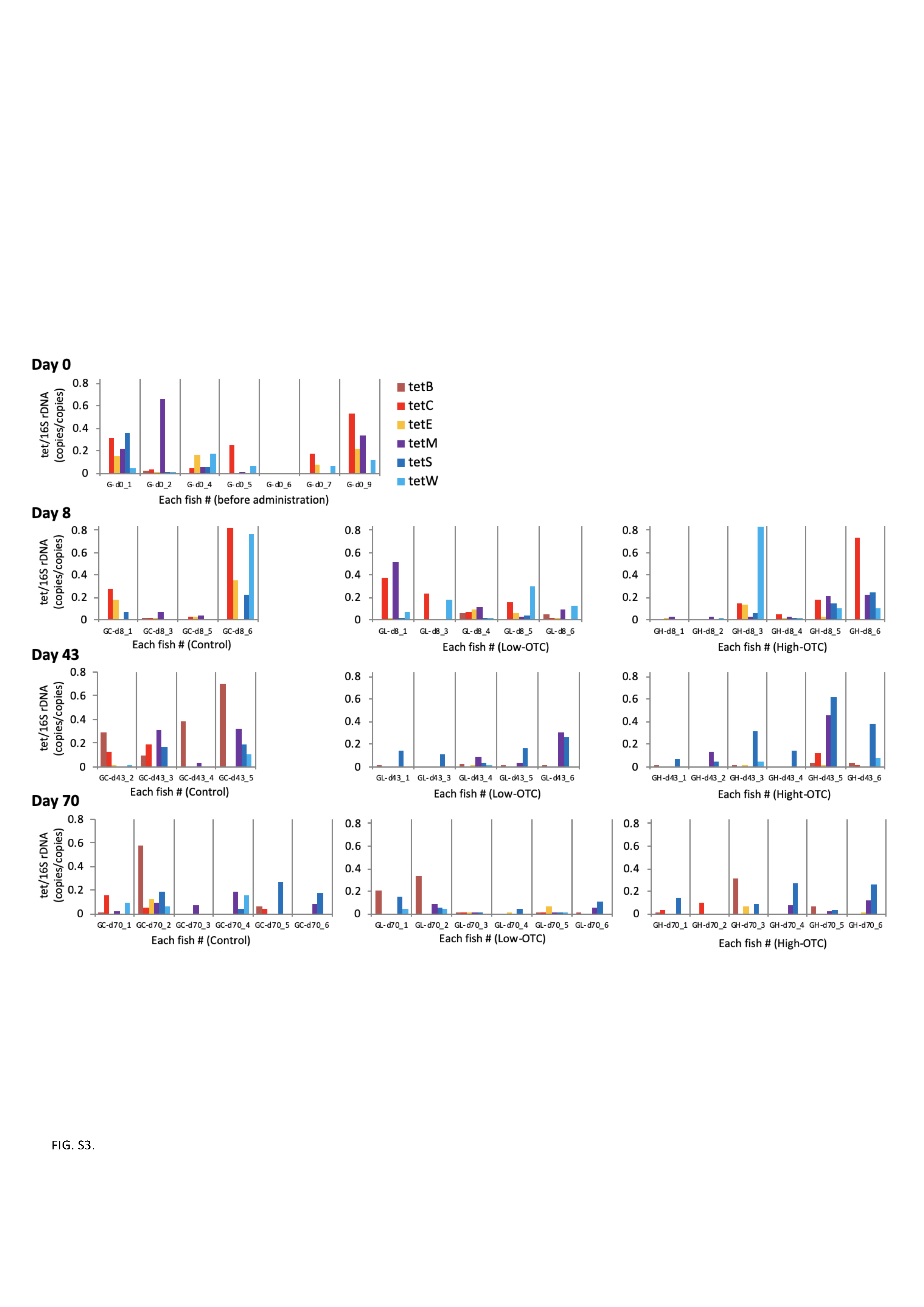

Supplement: FIGURE S3 — Copy numbers of tet genes in individual fish intestines; values were normalized to 16S rRNA gene copy number in the respective sample. Different tet genes are indicated by different bar colors. [file Image_3.tiff]
