# Supplementary material for: Tetracycline Resistance Gene Profiles in Red Seabream (Pagrus major) Intestine and Rearing Water After Oxytetracycline Administration
Source: Front Microbiol. 2020 Aug 4;11:1764. doi: 10.3389/fmicb.2020.01764 (PMC7417432; doi:10.3389/fmicb.2020.01764)
Supplement: TABLE S1 — List of primers used in this study for the quantitative PCR targeting of six tet genes and the 16S rRNA genes. [file Table_1.docx]

TABLE S1. List of primers used in this study for the quantitative PCR targeting of six *tet* genes and the 16S rRNA genes.

| Target gene | Primer ID | 5’⇒3’ sequence | Reference |
| --- | --- | --- | --- |
| 16S rRNA | Bact1369F | CGGTGAATACGTTCYCG | Suzuki *et al*. (2000) |
|  | Bact1492R | GGWTACCTTGTTACGACTT |  |
| TetB | TetB-F | TTGGTTAGGGGCAAGTTTTG | Ng *et al*. (2001) |
|  | TetB-R | GTAATGGGCCAATAACACCG |  |
| TetC | TetC-F | CTTGAGAGCCTTCAACCCAG | Ng *et al*. (2001) |
|  | TetC-R | ATGGTCGTCATCTACCTGCC |  |
| TetE | TetE-F | AAACCACATCCTCCATACGC | Ng *et al*. (2001) |
|  | TetE-R | AAATAGGCCACAACCGTCAG |  |
| TetM | qtetM-f | GCAATTCTACTGATTTCTGC | Tamminen *et al.* (2011b) |
|  | qtetM-r | CTGTTTGATTACAATTTCCGC |  |
| TetS | Tet(S)-FW | GAAAGCTTACTATACAGTAGC | Aminov *et al*. (2001) |
|  | Tet(S)-RV | AGGAGTATCTACAATATTTAC |  |
| TetW | Tet(W)-FW | GAGAGCCTGCTATATGCCAGC | Aminov *et al*. (2001) |
|  | Tet(W)-RV | GGGCGTATCCACAATGTTAAC |  |
